# Biologically Informed Machine Learning Prioritizes Dietary Supplements That Protect Neural Crest Cells from Ethanol-Induced Epigenetic Dysregulation and Developmental Impairment

**DOI:** 10.3390/ijms27010295

**Published:** 2025-12-27

**Authors:** Xiaoqing Wang, Miao Bai, Shuoyang Wang, Hongjia Qian, Jie Liu, Wenke Feng, Huang-ge Zhang, Xiaoyang Wu, Shao-yu Chen

**Affiliations:** 1 Department of Pharmacology and Toxicology, Health Sciences Center, University of Louisville, Louisville, KY 40292, USA; xiaoqing.wang@louisville.edu (X.W.);; 2 Alcohol Research Center, University of Louisville, Louisville, KY 40292, USA; 3 Department of Bioinformatics and Biostatistics, University of Louisville, Louisville, KY 40292, USA; 4 Department of Structural and Cellular Biology, School of Medicine, Tulane University, New Orleans, LA 70112, USA; 5 Department of Microbiology and Immunology, James Graham Brown Cancer Center, University of Louisville, Louisville, KY 40292, USA; 6 Robley Rex Veterans Affairs Medical Center, Louisville, KY 40292, USA; 7 Ben May Department for Cancer Research, University of Chicago, Chicago, IL 60605, USA

**Keywords:** artificial intelligence, biologically informed machine learning models, epigenetics, neural crest cells, fetal alcohol spectrum disorders (FASD)

## Abstract

The impairment of neural crest cells (NCCs) plays a pivotal role in the pathogenesis of fetal alcohol spectrum disorders (FASD). Epigenetic regulators mediate ethanol-induced disruptions in NCC development and represent promising targets for nutritional interventions. Here, we developed a biologically informed machine learning framework to predict nutritional supplements that modulate five key epigenetic regulators (*miR-34a*, DNMT3a, HDAC, *miR-125b*, and *miR-135a*) and mitigate ethanol’s adverse effects on NCCs. The optimized models demonstrated robust predictive performance and identified a number of nutritional supplements that could attenuate ethanol-induced NCC impairment, including resveratrol, vitamin B12, emodin, quercetin, and broccoli sprout-derived compounds. Our optimized models also revealed structural features that are critical for mitigating ethanol-induced NCC impairment through specific epigenetic mechanisms. These findings support predictive modeling as a tool to prioritize nutritional supplements for further investigation and the development of dietary strategies to prevent or reduce the risk of FASD.

## 1. Introduction

Fetal alcohol spectrum disorders (FASDs) represent a complex and enduring consequence of prenatal exposure to alcohol during pregnancy [1,2]. It encompasses a broad range of adverse health implications in the developing fetus, including congenital defects, neurodevelopmental impairments, congenital anomalies, and growth retardation. FASD is the leading known cause of craniofacial dysmorphology and mental retardation in Western countries [1,3]. It has lifelong implications, as no cure is currently established [4,5]. Given the profound impact of FASD on public health and the absence of a recognized cure, there is an urgent need to develop effective preventive and interventive strategies for FASD [1,5,6].

The susceptibility of specific cell types to ethanol-induced cytotoxicity is a key factor contributing to FASD [7,8,9]. Among the cell populations most vulnerable to ethanol’s harmful effects are neural crest cells (NCCs) [9,10,11,12]. These multipotent cells can differentiate into various cell types and contribute to the formation of structures such as cartilage, connective tissues, and the skeletal components of the head and face [13,14,15]. Disruptions in NCC development can lead to neurocristopathies, a group of developmental disorders, including craniofacial abnormalities, hearing impairments, and heart defects [16]. Research has demonstrated that ethanol exposure can interfere with craniofacial development by disrupting several critical processes in cranial NCC development, such as induction, migration, differentiation, and survival [1].

Previous research in our laboratory and others has demonstrated that epigenetic regulators, including microRNA (miR)-34a, *miR-125b*, *miR-135a*, DNA methyltransferases (DNMT3a), and histone deacetylases (HDAC), are involved in ethanol-induced impairment in NCC migration, differentiation, and survival [17,18,19,20,21]. For example, Fan et al. [18,22] found that *miR-34a* inhibitors restored the expression of autophagy-related 9A (Atg9a) target and diminished the upregulation of E-cadherin1 to restore epithelial–mesenchymal transition and migration in ethanol-exposed NCCs [18,22]. Moreover, Chen et al. [17] also found that *miR-125b* mimics significantly decreased the protein expression of PUMA/Bak1 and caspase-3 activation, diminishing ethanol-induced apoptosis in NCCs and growth retardation in mouse embryos. *miR-135a* mimics also protected against ethanol-induced apoptosis in NCCs and craniofacial defects in the zebrafish model of FASD by inhibiting the activation of the p38 MAPK/p53 pathway [21]. Li et al. also found that diminishing the upregulation of DNMT3a could attenuate ethanol-induced apoptosis by reducing hypermethylation at the promoter regions of anti-apoptotic genes [19]. Additionally, several HDAC inhibitors have been shown to increase histone acetylation at the promoter regions of the Bcl-2 or Snail1 gene, resulting in the upregulation of Bcl-2 or Snail1 and a reduction in apoptosis in ethanol-exposed NCCs [20,23]. This suggests that these epigenetic regulators could serve as potential targets for therapeutic prevention of FASD.

Nutritional supplements have shown potential in preventing and treating FASD [19,20,23,24,25,26]. For example, folic acid can reduce ethanol-induced effects, including growth retardation and neuronal loss [27]. Similarly, choline has been found to improve cognitive function and reduce behavioral issues in animal models of FASD [28]. In addition, zinc supplementation has demonstrated protective effects against fetal malformations and cognitive impairments in offspring exposed to ethanol [29,30]. Furthermore, antioxidants play a key role in neutralizing oxidative stress caused by ethanol, thus supporting fetal development [31]. Our research also indicates that antioxidants, such as superoxide dismutase (SOD), catalase mimetic EUK134, N-acetylcysteine, Nrf2 inducers such as tBHQ, and epigenetic modulators such as sulforaphane, can mitigate ethanol’s harmful effects on NCCs and embryos [19,20,23,24,25,26]. These findings underscore the importance of a comprehensive nutritional approach to FASD prevention and intervention.

Epigenetic regulators may be affected by a wide range of structural and chemical modifications of dietary supplements [19,20]. Identifying the key structural features of the dietary supplements or nutrients that contribute to the modulation of epigenetic regulators could significantly enhance the selection of dietary supplements and nutrients for protecting against FASD. However, experimental identification of all potential structural features targeting these epigenetic regulators is costly, time-consuming, and labor-intensive. Therefore, leveraging computational approaches, particularly advances in artificial intelligence, is essential for efficiently discovering and prioritizing promising therapeutic agents for FASD intervention.

Artificial intelligence, including machine learning, can efficiently process biological network data, enabling computational screening of hundreds of candidates by modeling network structures to interpret mechanisms, thus offering significantly higher efficiency in predicting the therapeutic potential of dietary supplements and nutrients for specific diseases or disorders [32,33,34]. Machine learning algorithms are increasingly proving valuable in overcoming categorization challenges and identifying criteria to rank potential nutrients or therapeutic strategies [35,36,37,38]. However, despite growing interest in artificial intelligence applications, the research at the intersection of epigenetics and machine learning remains notably underexplored. Existing machine learning studies in FASD have primarily relied on phenotype-driven and clinical data, including behavioral, neurocognitive, demographic, imaging, and medical record information [39,40,41,42]. These studies primarily focus on postnatal diagnostic classification and rely on observable symptoms or retrospective exposure data. In contrast, few studies have investigated the underlying biological mechanisms, particularly those involving epigenetic regulation. In addition, the limited integration of biological knowledge into machine learning models has contributed to a lack of interpretability, thereby restricting their translational potential and broader applications in biomedical research. Addressing these gaps requires biologically informed models that connect molecular mechanisms with relevant phenotypes to enhance understanding of FASD pathogenesis.

In this study, biologically informed machine learning models were developed by integrating multiple machine learning algorithms with key epigenetic regulators, including *miR-34a*, DNMT3a, HDAC, *miR-125b*, and *miR-135a*, to prioritize dietary supplements or nutrients for preventing or mitigating the adverse effects of ethanol on NCCs. Our optimized models successfully predicted and identified a number of dietary supplements and nutrients that may attenuate ethanol-induced impairment in NCC development by targeting specific epigenetic regulators. In addition, these models revealed key structural features potentially associated with the mitigation of ethanol’s adverse effects in these dietary supplements or nutrients and demonstrated that compounds with distinct structural characteristics can attenuate ethanol-induced disruption in NCC development by modulating different epigenetic regulators. Overall, the biologically informed machine learning models provide an effective and reliable strategy for predicting and prioritizing dietary supplements or nutrients to alleviate the impact of prenatal ethanol exposure on NCCs and reduce the prevalence of FASD.

## 2. Results

### 2.1. Development and Optimization of the Biologically Informed Machine Learning Models for Each Epigenetic Regulator Module

**Figure 1:**
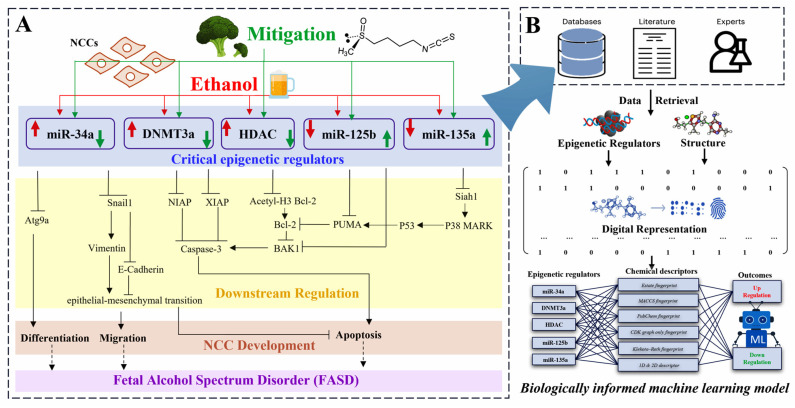
Overview of the biologically informed machine learning framework with a bio-centric interpretability scheme based on the mode of action of therapeutic agents in preventing ethanol-induced impairment in NCCs. (**A**) Ethanol disrupts key epigenetic regulators, including *miR-34a*, DNMT3a, HDAC, *miR-125b*, and *miR-135a*, leading to impairment of NCC differentiation, migration, and survival through pathways involving Atg9a, Snail1, EMT, Bcl-2, PUMA, Siah1, p38 MARK, and P53, leading to developmental defects associated with prenatal alcohol exposure and FASD. In contrast, dietary supplements may exert protective effects by modulating these epigenetic regulators, restoring the disrupted pathways, and mitigating ethanol-induced impairments in NCC differentiation, migration, and survival. This figure was constructed primarily based on previous findings [17,18,21,22,23,26,43,44,45,46] and reviews [1] from our laboratory, and served as the conceptual foundation for the subsequent computational modeling. Vertical red arrows indicate the direction of ethanol-induced changes in epigenetic regulators, with upward arrows representing upregulation and downward arrows representing repression. Similarly, vertical green arrows depict the regulatory effects of therapeutic agents, indicating upregulation or repression of epigenetic regulators in NCCs. Additionally, the dashed arrows depict that ethanol-induced impairments in NCC differentiation, migration, and survival may contribute to FASD. T-shaped arrows represent inhibition. (**B**) The biologically informed machine learning framework integrates literature, databases, and expert-curated data to retrieve key epigenetic regulators and the chemical structure information of dietary supplements or nutrients, and is refined using experimentally validated targets characterized in our laboratory (as shown in (**A**)), enabling targeted prediction of compound-induced regulatory effects. After digital encoding, a machine learning model is trained to predict whether dietary compounds can upregulate or downregulate specific epigenetic regulators. This allows for in silico screening of potential protective agents against ethanol-induced epigenetic disruptions and NCC impairment.

**Figure 2:**
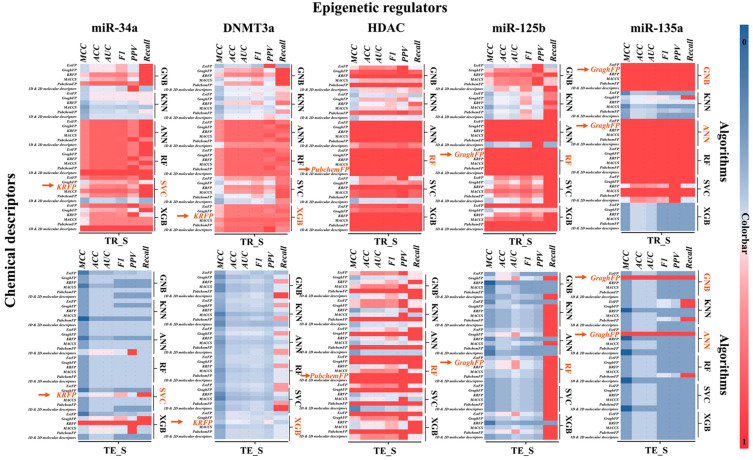
Performance evaluation of the developed machine learning models based on MCC, ACC, AUC, F1, PPV, and Recall metrics for both the training sets (TR_S) and external testing sets (TE_S). The left *Y*-axis denotes the different algorithms, molecular descriptors, and model performance parameters, while the right *Y*-axis distinguishes results between the training and testing sets. The *X*-axis represented the five epigenetic regulator modules (*miR-34a*, DNMT3a, HDAC, *miR-125b*, and *miR-135a*). Colorbar: The color gradient from blue (value of 0) to red (value of 1) represents the absolute values of each performance metric, with red indicating higher values and superior model performance. Higher metric values reflect improved model parameters and enhanced model performance. Orange arrows highlight the optimal model for each module, providing a clear and intuitive visualization of the selection process compared to other models. ACC: Accuracy; AUC: Area under the receiver operating characteristic curve; PPV: Positive predictive value; Recall: Ratio of correct positive predictions to actual positives; F1: F1 Score; MCC: Matthews correlation coefficient; ANN neural networks-multilayer perceptron; KNN: K-neighbors classifier; GNB: Gaussian Naive Bayes; RF: Random forest; SVC: Support vector machine; XGB: Extreme gradient boosting decision tree; PubchemFP: Publicly available fingerprint descriptors capturing presence of substructures; MACCS: 166-bit structural key fingerprints, commonly used in cheminformatics; KRFP: Klekota–Roth fingerprint, a substructure-based fingerprint encoding the presence of predefined chemical fragments; GraphFP: Graph-based fingerprints capturing topological features; EstFP: Electrotopological state indices reflecting electronic and topological properties.

The biologically informed machine learning models were developed using chemical structure data of dietary supplements and nutrients with known or predicted interaction with key epigenetic regulators (*miR-34a*, DNMT3a, HDAC, *miR-125b*, and *miR-135a*) curated from extensive literature [17,18,21,22,23,26,43,44,45,46] and comprehensive data retrieval from multiple databases, and further refined using experimentally validated targets characterized in our laboratory (Figure 1). This biologically grounded design enables the machine learning models to predict and prioritize potential dietary supplements and nutrients that can mitigate ethanol-induced impairment in neural crest cell (NCC) development and to elucidate their underlying molecular mechanisms. In this study, 180 biologically informed machine learning models were developed using six different machine learning algorithms and six types of chemical descriptors, avoiding the limitations of relying on only a single algorithm or descriptor class (Figure 2). The results showed that the most effective algorithm and chemical descriptor varied across different epigenetic regulators. For example, the XGB and RF algorithms outperformed others in the DNMT3a and HDAC modules, while the SVC, RF, GNB, and ANN algorithms performed well in the microRNA modules (*miR-34a*, miR-125a, and miR-135b) (Figure 2). In terms of descriptors, KRFP was the most effective descriptor for modeling the *miR-34a* and DNMT3a modules, capturing key substructural features potentially relevant to these targets. In contrast, GraphFP, which encodes molecular graph topology, yielded the best performance in the *miR-125b* module, while ExtFP, a type of extended fingerprint descriptor, was essential for accurately modeling *miR-135a*. In the HDAC module, the PubchemFP descriptor covering broad fragment-level information was effective for characterizing compounds’ structures. Combining the most effective machine learning algorithms with carefully selected molecular descriptors and fingerprints facilitates the development of optimal predictive models for distinguishing various epigenetic regulators.

**Figure 3:**
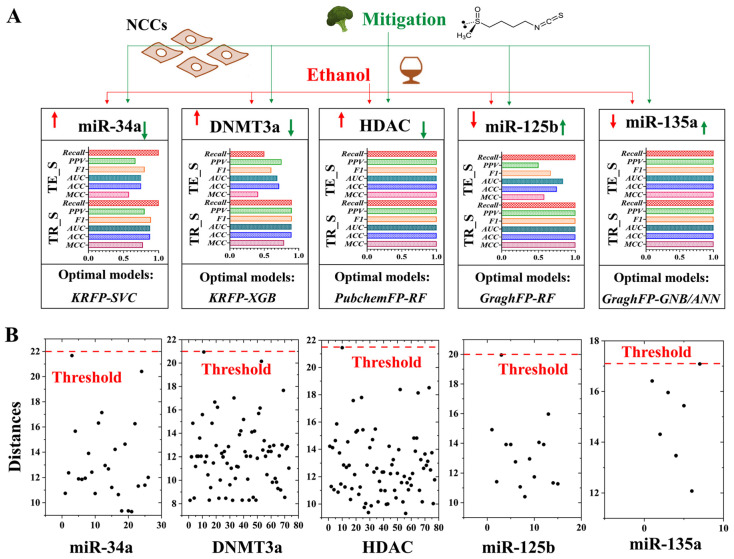
Performance evaluation and application domains of the optimized machine learning framework for predicting epigenetic regulators. (**A**) Histograms illustrated the values of performance metrics of the optimal models across the five modules: *miR-34a*, DNMT3a, HDAC, *miR-125b*, and *miR-135a*. Red upward arrows denote ethanol-induced upregulation or activation of regulators/biological processes; red downward arrows indicate ethanol-induced downregulation or inhibition of regulators/biological processes. Green downward arrows represent the mitigation of ethanol-induced adverse effects through downregulating or inhibiting the epigenetic regulators/biological processes, while green upward arrows represent the mitigation of ethanol-induced adverse effects through upregulating or activating epigenetic regulators/biological processes. (**B**) The Euclidean distance plot illustrates the application domain of the model. The red dashed line denotes the threshold for domain applicability, while the vertical positions of the black dots represent the Euclidean distances for individual compounds.

The best-performing biologically informed machine learning models were selected through a rigorous ten-fold cross-validation process, utilizing multiple performance metrics, including accuracy (ACC), area under the receiver operating characteristic curve (AUC), positive predictive value (PPV), ratio of correct positive predictions to actual positives (Recall), F1 score, and Matthews correlation coefficient (MCC) scores, across both the training and external testing sets. This process led to the identification of the most suitable models for each epigenetic regulator module. Overall, the optimal biologically informed machine learning models consistently achieved an accuracy of over 75% (Figure 3A). This high accuracy highlights the effectiveness of the models in capturing relevant biological signals and chemical features. In detail, for the *miR-34a* module, the KRFP-SVC model was identified as the optimal choice, offering balanced performance across all metrics. Similarly, for the *miR-125b* module, the GraphFP-RF model, which demonstrates strong reliability, was chosen. For the *miR-135a* module, the GraghFP-GNB/ANN model stood out, with both the GNB and ANN algorithms delivering exceptional performance (Figure 3A). Additionally, the KRFP combined with the XGB algorithm showed strong performance in the analysis of DNMT3a modules. In contrast, the PubchemFP-RF model outperformed others in the HDAC module analysis (Figure 3A). Overall, the results demonstrate that these models exhibit high accuracy, sensitivity, and specificity, making them powerful tools for accurately identifying and characterizing key epigenetic regulator modules and their potential utility in advancing the screening of epigenetic therapeutic strategies. Notably, the developed models are specifically designed for application in the biological domain, with a particular focus on predicting and analyzing epigenetic regulation. Euclidean distance analysis revealed that almost all dietary supplements fell within the applicability domain range of the models and were accurately predicted. This suggests that the models provide reliable and accurate predictions, as most data points were within the predefined applicability range (Figure 3B). Normalized distance plots further confirmed the models’ sustained accuracy.

### 2.2. Developed Machine Learning Models Predicted Various Dietary Supplements or Nutrients with the Potential to Mitigate Ethanol-Induced Impairment in NCCs by Targeting Epigenetic Regulators

**Figure 4:**
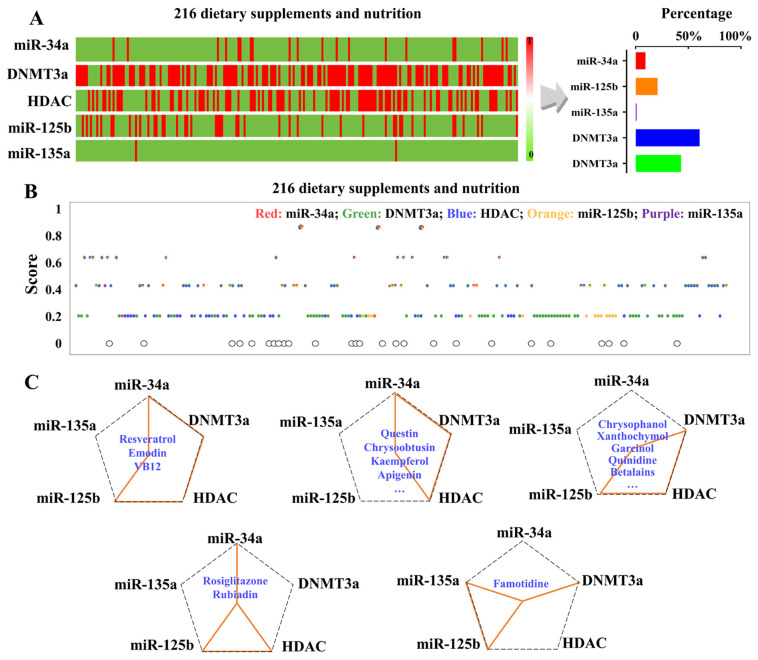
Prediction workflow of key epigenetic regulator-based optimal ML models. (**A**) Heatmaps (**left**) display the predictions for 216 candidate dietary supplements or nutrients. Red indicates that the dietary supplement or nutrient is predicted to have a mitigating effect through a given epigenetic regulator, while green denotes that the dietary supplement or nutrient is predicted to have no mitigating effects. The bar chart (**right**) illustrates the proportion of candidate dietary supplements or nutrients predicted to target each epigenetic pathway. Among these, DNMT3a-related regulation accounted for the largest proportion (60.65%), followed by HDAC (43.06%) and microRNAs (25.30%). Within the microRNA category, individual regulatory coverage was observed for *miR-34a* (9.26%), *miR-125b* (20.83%), and *miR-135a* (0.93%). Percentages were calculated separately for each regulator, and overlapping targets were included. (**B**) Ranking of dietary supplements or nutrients based on predicted mitigating potential against ethanol-induced adverse effects. The *Y*-axis shows the rank score, and the *X*-axis lists the 216 dietary supplements or nutrients. Dot colors represent the associated epigenetic regulator. Red: *miR-34a*; Green: DNMT3a; Blue: HDAC; Orange: *miR-125b*; Purple: *miR-135a*. White dots denote that dietary supplements or nutrients were predicted to have an insignificant mitigation effect on alcohol-induced effects by modulating the regulatory factors. Single-colored dots indicate that dietary supplements/nutrients are predicted to exert a mitigation effect through modulating a single epigenetic regulator (*miR-34a*, DNMT3a, HDAC, *miR-125b*, or *miR-135a*), while multi-colored dots reflect that the dietary supplements or nutrients are predicted to have a mitigation effect by modulating multiple epigenetic regulators. (**C**) The radar chart illustrates the epigenetic targets of selected high-potential compounds for mitigation. Each axis corresponds to one of the five key epigenetic regulators (e.g., *miR-34a*, DNMT3a, HDAC, *miR-125b*, or *miR-135a*). Each data point represents the prediction of each dietary supplement or nutrient with the corresponding epigenetic regulator. The orange lines indicate epigenetic regulatory targets that are predicted to be modulated by dietary supplements or nutrients. The blue text represents dietary supplements or nutrients that are predicted to exert mitigating effects through the indicated epigenetic regulators. An ellipsis indicates the presence of additional dietary supplements or nutrients beyond those explicitly labeled. The revised figure has been added.

As shown in Figure 4, the prediction dataset included 216 dietary supplements or nutrients, which were curated by removing missing data, anomalies, duplicates, or poorly defined structures. Of these 216 dietary supplements or nutrients, 25.30% of items, including citric acid, methyl salicylate, vitamin C, and caffeine, were identified as having a potential mitigating effect through the regulation of microRNA (Figure 4A). Specifically, around 9.26% of these items (such as salicylic acid, acetic acid, acetylsalicylic acid, ascorbic acid, and methyl salicylate) could mitigate the adverse effects of alcohol by downregulating *miR-34a*. Additionally, 20.83% of these items (such as citric acid, atorvastatin, lycopene, glutathione, vitamin A, cisplatin, and cortisol) and 0.93% of these items (such as hydrogen peroxide and progesterone) showed the potential to upregulate *miR-125b* or *miR-135a*-regulated pathways, respectively. Furthermore, 60.65% of these items in the prediction set, including n-Hexyl glucosinolate, sinigrin, luteolin, and glucocochlearin, demonstrated the potential to affect DNA methylation by downregulating the expression/activity of DNMT3a. Additionally, 43.06% of items, including boldine, betalains, apigenin, chrysophanol, rutin, and phycocyanobilin, had shown the potential to act as HDAC inhibitors to disturb the HDAC activity (Figure 4A).

Our developed models’ predictions provided valuable insights into how various dietary supplements or nutrients modulate key epigenetic regulators. Notably, resveratrol (3,5,4′-trihydroxy-trans-stilbene) emerged as a promising candidate for mitigating ethanol-induced adverse effects in NCCs (Figure 4). Specifically, our models predicted that resveratrol could reduce the expression or activity of *miR-34a*, DNMT3a, and HDAC, while increasing the expression of *miR-125b*, thereby decreasing ethanol-induced apoptosis and reducing ethanol-induced inhibition of differentiation and migration in NCCs, which may help prevent FASD (Figure 4B,C). In addition to resveratrol, vitamin B12 and emodin (6-methyl-1,3,8-trihydroxyanthraquinone) were also predicted to mitigate alcohol-induced adverse effects on NCC development and FASD by reducing the expression or activity of *miR-34a*, DNMT3a, and HDAC and increasing *miR-125b* expression (Figure 4B,C). Furthermore, several compounds, including kaempferol, apigenin, and chrysophanol, found in broccoli sprout extracts, were predicted to downregulate *miR-34a*, DNMT3a, and HDAC, and upregulate *miR-125b*, suggesting their potential to alleviate the negative effects of ethanol exposure on NCCs through modulation of these epigenetic regulators (Figure 4B,C). Other compounds, such as quercetin, vanguard xl-1, and chrysoobtusin, were also predicted to downregulate *miR-34a*, DNMT3a, and HDAC (Figure 4C), potentially mitigating the adverse effects of ethanol exposure. Furthermore, betalains, chrysophanol-9-anthrone, quinidine, garcinol, xanthochymol, and chrysophanol were predicted to downregulate DNMT3a and HDAC while upregulating *miR-125b* (Figure 4C). Rosiglitazone and rubiadin were predicted to downregulate *miR-34a* and HDAC while upregulating *miR-125b*, supporting their potential to mitigate ethanol-induced damage. Moreover, famotidine was predicted to downregulate DNMT3a and upregulate *miR-135a* and *miR-125b* (Figure 4C). Overall, the predictions from our models offer valuable insights into how various dietary supplements and nutrients may regulate epigenetic regulators to alleviate ethanol-induced adverse effects and their potential mechanisms.

### 2.3. Key Structural Features in Predicted Dietary Supplements and Nutrients That Contribute to Epigenetic Regulation and the Mitigation of Ethanol’s Adverse Effects Were Identified Using the Developed Machine Learning Models

**Figure 5:**
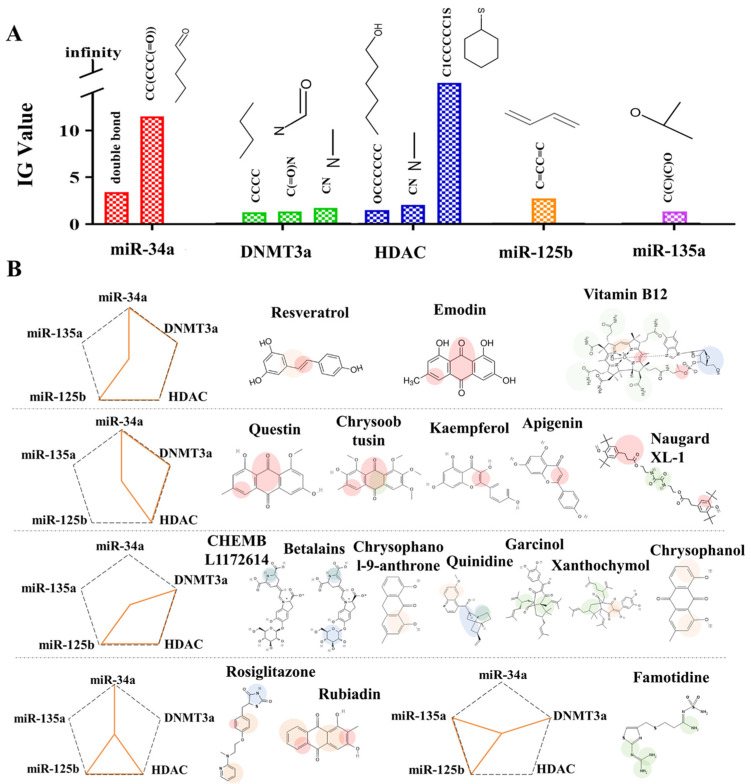
Structural features associated with the mitigation of ethanol-induced epigenetic disruption. (**A**) Key structural features identified through visual inspection as critical for alleviating ethanol-induced alterations in *miR-34a*, DNMT3a, HDAC, *miR-125b*, and *miR-135a* modules. (**B**) Representative structural profiles of top-ranked dietary supplements or nutrients predicted to modulate specific epigenetic regulators. Colored circles denote the epigenetic regulators targeted by these dietary supplements or nutrients, with red circles for *miR-34a*, green circles for DNMT3a, blue circles for HDAC, orange circles for *miR-125b*, and purple circles for *miR-135a*. The orange lines indicate epigenetic regulatory targets that are predicted to be modulated by dietary supplements or nutrients.

Next, we utilized the developed machine learning models to elucidate the mechanisms by which the predicted dietary supplements and nutrients mitigate the adverse effects of alcohol by targeting epigenetic regulators. The models revealed that specific substructures, including double bonds, CC(CCC(=O)), C=CC=C, C(C)(C)O, C(=O)N, CCCC, CN, OCCCCCC, and C1CCCCC1S, were potentially associated with alleviating ethanol-induced disruption in NCC development and FASD (Figure 5A). Dietary supplements or nutrients containing double bonds or CC(CCC(=O)) structures were more likely to mitigate ethanol-induced impairment in NCC migration and differentiation by downregulating *miR-34a* expression. Meanwhile, those containing C=CC=C and C(C)(C)O structures were more likely to alleviate ethanol-induced apoptosis in NCCs through the upregulation of *miR-125b* and *miR-135a* (Figure 5A). Additionally, dietary supplements or nutrients containing C(=O)N/CCCC and CN/OCCCCCC/C1CCCCC1S structures were predicted to reduce ethanol-induced apoptosis in NCCs by inhibiting DNMT3a and HDAC, respectively (Figure 5A). Notably, resveratrol, VB12, and emodin, which were predicted to have a high potential for mitigating ethanol-induced adverse effects in NCCs, likely exert their protective effects due to the presence of double bonds, C(=O)N and benzene rings in their structures, as supported by the feature importance analysis of the machine learning models (Figure 5B). Additionally, the double bonds and CC(CCC(=O)) structures in quercetin, chrysoobtusin, kaempferol, apigenin, and rubiadin may play a critical role in regulating *miR-34a*, thereby alleviating the alcohol-induced adverse effect. The presence of C(=O)N and CN structures in naugard xl-1, chrysoobtusin, garcinol, xanthochymol, and famotidine suggests their potential to mitigate ethanol’s effects on NCCs by targeting DNMT3a. Furthermore, betalains, quinidine, and rosiglitazone were predicted to downregulate HDAC, possibly due to the presence of OCCCCCC and CN structures. Moreover, the C=CC=C structure in chrysophanol, rosiglitazone, rubiadin, and the C(C)(C)O structure in resveratrol likely contribute to the upregulation of *miR-125b* and *miR-135a*, helping to counteract the ethanol-induced effects on NCCs.

## 3. Discussion

**Figure 6:**
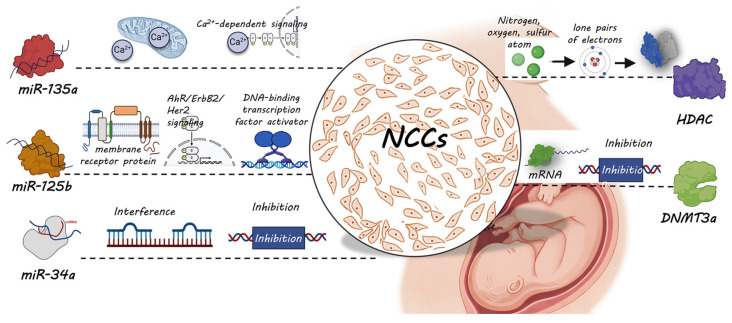
Schematic illustration of epigenetic mechanisms by which representative dietary supplements or nutrients mitigate ethanol-induced impairments in NCC development, potentially preventing alcohol-associated developmental defects and FASD.

Epigenetic regulation, such as DNA methylation, histone modifications, and microRNAs, plays a crucial role in both alcohol-induced injury and its mitigation [17,21]. Epigenetic regulators, such as *miR-34a*, DNMT3a, HDAC, *miR-125b*, and *miR-135a*, have been identified as potential therapeutic targets for preventing FASD by regulating key cellular processes such as differentiation, proliferation, and apoptosis of NCCs [17,18,19,20,21]. However, identifying protective dietary supplements or nutrients for the intervention of FASD through traditional experimental screening is labor-intensive and time-consuming. In this study, we developed and implemented a biologically informed machine learning framework that integrates multiple machine learning algorithms to identify potential dietary supplements and nutrients capable of mitigating ethanol-induced disruptions in NCC development and FASD. Our computational approach enables high-throughput, mechanistically informed predictions based on compound structure and epigenetic regulatory activity (Figure 6).

By leveraging six different machine learning models, namely ANN, KNN, GNB, RF, SVC, and XGB, we systematically evaluated model performance across various molecular fingerprint inputs, including KRFP, PubChem, and graph-based representation fingerprints. Our analysis demonstrated that specific combinations of model and fingerprint type yielded superior predictive performance. In particular, models such as KRFP-SVC, KRFP-XGB, PubchemFP-RF, GraphFP-RF, and GraphFP-GNB/ANN consistently showed high accuracy in characterizing the regulatory states of five key epigenetic targets: *miR-34a*, DNMT3a, HDAC, *miR-125b*, and *miR-135a*. These epigenetic factors were chosen based on their established roles in NCC development and vulnerability to ethanol-induced dysregulation [17,18,19,20,21,22,23]. Their accurate modeling suggests that our machine learning framework not only captures the structural features of candidate compounds but also reflects their biological relevance in modulating pathways critical for NCC development.

A key finding of this study was the demonstration of model robustness in predicting dietary supplements and nutrients that may mitigate ethanol-induced disruption in NCC development and reduce the risk of FASD. Using our optimized ensemble of machine learning models, we identified several compounds with strong predicted protective effects. Among these, resveratrol and vitamin B12 stood out due to their well-documented antioxidant and neuroprotective properties [47,48,49], which align with mechanisms implicated in counteracting ethanol-induced oxidative stress and epigenetic dysregulation [1,26,50]. In addition, we identified a diverse group of phytochemicals and bioactive plant extracts, including emodin, quercetin, betalains, rubiadin, and various constituents of broccoli sprout extracts such as kaempferol, apigenin, and chrysophanol, that share structural motifs potentially associated with anti-inflammatory, antioxidant, or epigenetic modulatory activities. Many of these compounds have shown promise in other contexts, such as developmental toxicity or neurological protection [51,52,53,54,55,56,57,58], and our findings suggest that they may also have relevance for ethanol-induced teratogenesis. The convergence of computational predictions with biologically plausible and literature-supported candidates underscores the validity of our framework and its potential to guide the selection of compounds for experimental validation.

A significant strength of our machine learning framework lies in its ability not only to identify candidate compounds but also to provide insights into their potential mechanisms of action. By analyzing the common structural features among the predicted compounds, we identified key molecular substructures, such as double bonds, CC(CCC(=O)), C=CC=C, C(C)(C)O, C(=O)N, CCCC, CN, OCCCCCC, and C1CCCCC1S, as potential contributors to their protective effects. These structures align with known biological activities affecting epigenetic regulators, suggesting mechanistic relevance. Among these, structural features such as the double bond and the CC(CCC(=O)) motif were identified as being strongly associated with the predicted inhibition of *miR-34a*, a key miRNA known to impair NCC migration and differentiation following ethanol exposure [18,59]. These fragments were associated with predicted *miR-34a* inhibition and may potentially act via the p53-mediated pathway, a transcriptional activator of *miR-34a* with well-conserved binding sites in its promoter region [60,61,62]. However, this proposed mechanism remains hypothetical and requires experimental validation. Compounds such as ursodeoxycholic acid (UDCA) and its epimer chenodeoxycholic acid (CDCA), which contain a double bond and a CC(CCC(=O)) structure, have been shown to suppress *miR-34a* expression via p53 inhibition [63,64]. Additionally, sulforaphane (SFN), containing sulfur-oxygen double bonds, carbon-nitrogen double bonds, and sulfur double bonds, has demonstrated the ability to reverse the increased *miR-34a* expression and protect against apoptosis [65]. Other structural features, such as C=CC=C and C(C)(C)O were potentially associated with the upregulation of miRNAs with protective roles, including *miR-125b* and *miR-135a*, which have been demonstrated to mitigate ethanol-induced apoptosis in NCCs [17,21]. Aminoflavone and carvedilol, in which C=CC=C acts as one of the critical structural features, were found to upregulate *miR-125b* by regulating the expression of the membrane receptor protein (e.g., α6-integrin) and modulating signaling pathways, including the aryl hydrocarbon receptor (AhR) and ErbB2/Her2 pathway or DNA-binding transcription factor activator activity (such as suppression of ITGA6) that control cell proliferation, epithelial-to-mesenchymal transition (EMT), differentiation, and apoptosis [66,67,68,69,70].

In terms of the regulation of DNA methylation, this study revealed that dietary supplements with C(=O)N, CCCC, and CN structures were potentially associated with the suppression of DNMT3a, a DNA methyltransferase involved in abnormal gene silencing during ethanol exposure [19]. For example, bexarotene containing CCCC was found to reduce the expression of DNMT3a mRNA [71]. Moreover, azacitidine, which has C(=O)N, CCCC, and CN structures, could inhibit the binding of DNMT3a protein to specific promoters [72]. Notably, these structures (C(=O)N, CCCC, and CN) are also common in known DNMT inhibitors, such as decitabine, zebularine, SGI-1027, fazarabine, decitabine, and nanaomycin A [73,74,75,76]. Additionally, vitamin B12, which contains both C(=O)N and CN, has been experimentally validated to reduce ethanol-induced developmental toxicity and improve cognitive outcomes [77,78], supporting our computational predictions.

Our models also revealed that the inhibition of histone deacetylases (HDACs), another major epigenetic regulator, was potentially associated with CN, OCCCCCC, and C1CCCCC1S structures. These structures are present in known HDAC inhibitors [79]. For instance, the CN structure is found in Trichostatin A (TSA), LBH589 (panobinostat), suberoylanilide hydroxamic acid (SAHA), trapoxin (TPX), MS 275 (entinostat), and FK228 (romidepsin). The OCCCCCC structure is present in MS 275, TSA, SAHA, and MS 275, and PXD101 (belinostat) contains the C1CCCCC1S structure [79,80,81,82]. The mechanistic basis for their inhibitory effects may involve the chelation of zinc and other metal ions in HDAC active sites through lone-pair donation by nitrogen, oxygen, or sulfur atoms in these structures [79,83,84], ultimately inhibiting HDAC activity. Our previous studies have shown that SFN, which contains CN structures, could alleviate ethanol-induced apoptosis in NCCs by reducing HDAC expression and activity [19,20].

Importantly, the structural characteristics of dietary supplements or nutrients with a high predictive rank score also aligned with the results of permutation importance analysis of structural features in different epigenetic regulators’ modules. For example, salicylic acid, acetic acid, acetylsalicylic acid, ascorbic acid, methyl salicylate, and other dietary supplements and nutrients could potentially decrease *miR-34a* expression. The majority of these supplements and nutrients have a double bond and a CC(CCC(=O)) structure. Atorvastatin, lycopene, vitamin A, and cortisol, which feature C=CC=C, were predicted to upregulate *miR-125b* expression. Compounds with the C(C)(C)O structure, such as progesterone and hydrogen peroxide, may upregulate the pathways modulated by *miR-135a*. Additionally, n-Hexyl glucosinolate, sinigrin, luteolin, and glucocochlearin, which contain the C(=O)N, CCCC, and CN structures, were predicted to mitigate the effects of ethanol by suppressing the expression or activity of DNMT3a. Furthermore, boldine, betalains, apigenin, chrysophanol, rutin, and phycocyanobilin containing CN, OCCCCCC, and C1CCCCC1S structures were predicted to inhibit HDAC (Figure 6)**.** Overall, our findings suggest that the most frequently observed and biologically active structures, double bonds and CN groups, are consistently associated with compounds that mitigate ethanol-induced epigenetic dysregulation and impairment in NCCs. These insights may help inform and guide future experimental work for rationally designing dietary supplements or therapeutic compounds to prevent FASD. Future investigations should prioritize these structural motifs and explore their broader implications in epigenetic modulation and developmental neuroprotection.

## 4. Materials and Methods

### 4.1. Construction of a Representative Dataset of Epigenetic Regulators Through Data Retrieval and Preprocessing

To develop biologically informed machine learning models for predicting and prioritizing potential dietary supplements and nutrients that prevent ethanol-induced impairment in NCC development and to elucidate their underlying mitigation mechanisms, five key epigenetic regulators, including *miR-34a*, DNMT3a, HDAC, *miR-125b*, and *miR-135a*, were selected and organized based on existing literature [17,18,21,22,23,26,43,44,45,46]. We initially retrieved thousands of in chemico, in vitro, and in vivo data points from PubChem, Toxicity Forecasting (ToxCast), and Comparative Toxicogenomics Database (CTD) to construct the datasets (Figure 1), characterizing the key epigenetic regulators and chemical characteristics relationship, which served as the basis for subsequent machine learning modeling. In the simplified biological framework, relationships among key epigenetic regulators, including *miR-34a*, DNMT3a, HDAC, *miR-125b*, and *miR-135a*, and their downstream effects on the migration, differentiation, and survival of NCCs were illustrated using color coding, providing a distinct categorization of links (Figure 1A). In total, approximately 200 samples were used for training and 50 samples for testing across these five epigenetic regulators. And each key epigenetic regulator module was classified as either upregulated/agonistic (denoted as 1) or downregulated/antagonistic (denoted as 0) (Figure 1B). Chemical records with ambiguous classifications or belonging to multiple categories were excluded, as were inorganic compounds, salts, and mixtures. Two-dimensional chemical structures were obtained from the US EPA Aggregated Computational Toxicity Resource (ACToR) database [85] and cross-referenced with the PubChem database to ensure consistency and accuracy.

### 4.2. Preparation and Balancing of Molecular Input Data for the Development of Biologically Informed Machine Learning Models

The molecular information for each compound was obtained through quantitative calculations of chemical descriptors, providing physicochemical properties ranging from topological to electrostatic terms. The input data for biologically informed machine learning model development comprised five categories of molecular fingerprints, including the Estate fingerprint (EstFP), MACCS fingerprint (MACCS), PubChem fingerprint (PubchemFP), CDK Graph Only fingerprint (GraphFP), and Klekota–Roth fingerprint (KRFP), along with 1613 categories of 1D and 2D molecular descriptors. To address the data imbalance between active and inactive samples, the synthetic minority oversampling technique was employed. After screening, the curated dataset, comprising quantitative chemical descriptors of individual compounds and their corresponding binary regulatory classification (upregulated/agonistic and downregulated/antagonistic) of key epigenetic regulators (Figure 1), was randomly split into training and independent testing sets in a 4:1 ratio to enable model validation.

### 4.3. Development and Validation of Biologically Informed Machine Learning Models

To assess which learning paradigm generalizes to unseen dietary supplements or nutrients, we benchmarked six different machine learning algorithms to develop models, including the multilayer perceptron -based neural networks (ANN)for capturing complex, non-linear relationships; k-nearest neighbors (KNN), a distance-based non-parametric classifier; Gaussian Naive Bayes (GNB), a probabilistic classifier; random forest (RF), an ensemble of decision trees for robust predictions; support vector machine (SVC), which identifies the optimal hyperplane for class separation; and extreme gradient boosting (XGB), an efficient gradient boosting algorithm. Considering the complex relationships between chemical structural features and epigenetic regulatory outcomes, these diverse algorithms, both linear and non-linear models, distance-based methods, probabilistic classifiers, ensemble methods, and deep learning architectures were included in our predictive framework. These models were evaluated using a variety of performance metrics to ensure robustness and predictive reliability. All procedures were implemented using the Scikit Learn package (sklearn) and the Xgboost package (Python 3.8). To evaluate the robustness and generalizability of the models, a ten-fold cross-validation was used as an internal check during model training [86,87]. This protocol helps quantify potential overfitting and provides a more reliable estimate of predictive performance.

### 4.4. Performance Evaluation and Optimization of the Developed Biologically Informed Machine Learning Models

To evaluate the performance and optimize the developed machine learning models, we calculated model evaluation metrics, including True Positives (*TP*), True Negatives (*TN*), False Positives (*FP*), and False Negatives (*FN*) from the confusion matrix. Additional performance metrics, including Accuracy (*ACC*), Area Under the Receiver Operating Characteristic Curve (*AUC*), Positive Predictive Value (*PPV*), Ratio of Correct Positive Predictions to Actual Positives (*Recall*), F1 score, and Matthews Correlation Coefficient (*MCC*), were assessed (Equations (1)–(6)) [88]. In addition, to determine the applicability range of the developed models, we utilized the Euclidean distance-based approach [89,90]. The distance values were calculated and normalized to a range of 0 to 1 and used to determine whether new compounds fell within the established domain of the machine learning models [91].

(1) $$\mathrm{ACC}=(\mathrm{TP}+\mathrm{TN})\;({\mathrm{TP}+\mathrm{TN}\;+\mathrm{FP}+\mathrm{FN})}^{-1}$$

(2) $$\mathrm{AUC}=\int_{0}^{1}\mathrm{TPR}\;({\mathrm{FPR}}^{-1}\;(x))dx$$

(3) $$\mathrm{PPV}=\mathrm{TP}\;{\left(\mathrm{TP}+\mathrm{FP}\right)}^{-1}$$

(4) $$\mathrm{Recall}=\mathrm{TP}\;{\left(\mathrm{TP}+\mathrm{FN}\right)}^{-1}$$

(5) $$F1=2\;\times \;(\mathrm{TP}\;{\left(\mathrm{TP}+\mathrm{FP}\right)}^{-1}\;\times \;\mathrm{Recall})\;{\left(\mathrm{TP}\;{\left(\mathrm{TP}+\mathrm{FP}\right)}^{-1}+\mathrm{Recall}\right)}^{-1}$$

(6) $$\mathrm{MCC}=(\mathrm{TP}\;\times \;\mathrm{TN}\;-\;\mathrm{FP}\;\times \;\mathrm{FN})\;{\left(\left(\mathrm{TP}+\mathrm{FP}\right)\;(\mathrm{TP}+\mathrm{FN})\;(\mathrm{TN}+\mathrm{FP})\;(\mathrm{TN}+\mathrm{FN})\right)}^{-1/2}$$

### 4.5. Predicting and Prioritizing Potential Dietary Supplements and Nutrients by Using the Developed Machine Learning Models

Various dietary supplements and nutrients were screened from public literature to form the prediction sets. The structural information of these dietary supplements or nutrients was validated and supplemented using the PubChem database. Initial automated data cleansing removed isotopes, multicomponent chemicals, and compounds lacking structural data to ensure a unique and refined dataset. To enhance predictive performance, a Euclidean distance-based approach was applied to assess the similarity among the dietary supplements and nutrients. Subsequently, the impact of each dietary supplement or nutrient on individual epigenetic regulators was predicted and evaluated using the optimal machine learning models. As previously described, the downregulation of *miR-34a*, HDACs, or DNMT3a, or the upregulation of *miR-125b* or *miR-135a*, may mitigate ethanol-induced adverse effects [17,18,19,20,21]. Therefore, for the *miR-34a*, HDACs, and DNMT3a modules, if the node was predicted to be inactivated/downregulated, it was assigned a score of 1, and if it was predicted to be activated/upregulated, it was scored as 0. Conversely, for the *miR-125b* and *miR-135a* modules, if the node was predicted to be activated/upregulated, it was scored as 1, and if it was predicted to be inactivated/downregulated, it was scored as 0. The rank score of each dietary supplement or nutrient for each module was then calculated using an equal weight allocation strategy to obtain the rank score value [92]. Finally, leveraging the insights from the developed models, the dietary supplements or nutrients were clustered and ranked, and key mechanisms were elucidated.

### 4.6. Identification of Key Structural Features in Predicted Dietary Supplements and Nutrients That Contribute to Epigenetic Regulation and the Mitigation of Ethanol’s Adverse Effects

Identifying molecular functional groups and structural descriptors within the optimal biologically informed machine learning models can provide valuable insights into the role of structural features in these predicted highly potential dietary supplements and nutrients in modulating epigenetic regulators. To uncover the mechanisms by which these dietary supplements and nutrients mitigate the adverse effects of alcohol through targeting epigenetic regulators, the information gain (IG) method was employed to filter substructural fragments and identify key structural features using SARpy (SAR in Python 2.7). For the training set, SARpy employs a “string mining” approach to break chemical structures into fragments from SMILES notation and utilizes the “likelihood ratio” approach to identify fragments associated with specific outcomes [93,94].

## 5. Conclusions

This study focuses on the NCC population, a critical contributor to craniofacial, cardiac, and neural structures affected in FASD [1], to model and predict early-stage preventive strategies against ethanol-induced developmental defects. By targeting this embryonic window, our approach offers mechanistic insight into how ethanol disrupts key developmental processes and demonstrates how computational models can identify potential nutritional interventions aimed at mitigating these effects. Our biologically informed machine learning framework, which integrated five key epigenetic regulators, accurately identified dietary supplements and nutrients, such as resveratrol, vitamin B12, emodin, quercetin, and broccoli-sprout–derived compounds (kaempferol, apigenin, and chrysophanol), that may attenuate ethanol-induced NCC impairment. Many of these compounds are recognized for epigenetic modulation, antioxidant, anti-inflammatory, or neuroprotective properties, providing independent support for the model’s predictive accuracy. Structural analysis revealed specific molecular motifs, such as double bonds, CC(CCC(=O)), C=CC=C, C(C)(C)O, C(=O)N, CCCC, CN, OCCCCCC, and C1CCCCC1S, that may contribute to protective effects against ethanol-induced epigenetic dysregulation and NCC impairment.

Early prenatal intervention is biologically feasible and clinically meaningful, as demonstrated by the success of folic acid supplementation in preventing neural tube defects when administered before or shortly after conception [95]. Similarly, identifying dietary supplements that protect NCCs from prenatal alcohol exposure could guide early prenatal or preconception nutritional strategies, particularly for women at risk of alcohol exposure before pregnancy recognition. Moreover, epigenetic alterations induced by prenatal ethanol exposure can persist into postnatal life and may serve as molecular biomarkers for early detection and risk assessment. Understanding these durable epigenetic signatures also provides a foundation for exploring later-stage or postnatal interventions, as certain nutritional approaches (e.g., choline supplementation [95]) have shown promise in improving cognitive outcomes among children affected by FASD.

The present study serves as a hypothesis-generating, biologically informed prioritization framework, bridging computational prediction with mechanistic insight into ethanol-induced NCC impairment. However, several limitations remain. The current findings are based on in silico analyses, and direct in vitro and in vivo validation is necessary to confirm whether predicted compounds can causally ameliorate ethanol-induced developmental deficits. Validation studies are underway, including in vitro assays using human NCCs to evaluate effects on apoptosis, migration, and differentiation, and in vivo studies using zebrafish models to assess developmental and behavioral outcomes. Additionally, this study primarily emphasizes epigenetic mechanisms in NCCs during early development and does not capture the full complexity of FASD pathology. Future research will expand this framework to include additional cell types, developmental stages, and molecular readouts, such as DNA methylation patterns, microRNA networks, and neuroinflammatory markers in postnatal brain tissue. Integrating multi-omics data (transcriptomics and epigenomics) and physical and behavioral phenotypes from prenatal ethanol models will also enhance both the biological relevance and translational potential of the ML approach. Extending the framework to neural and glial systems may further link early embryonic disruptions to later neurobehavioral outcomes across the FASD continuum. Collectively, these directions will enhance the translational value of this framework and support its future application in early identification and nutritional intervention strategies for FASD.

## Data Availability

The original contributions presented in this study are included in the article/supplementary material. Further inquiries can be directed to the corresponding author(s). The dataset and code of this study are available at GitHub (version 3.5): https://github.com/Shawn-UL/Epi_ML_FASD_XW.git (accessed on 29 July 2025).

## Supplementary Materials

The following supporting information can be downloaded at: https://www.mdpi.com/article/10.3390/ijms27010295/s1.

## Abbreviations

The following abbreviations are used in this manuscript:

FASDs	Fetal Alcohol Spectrum Disorders
NCCs	Neural crest cells
*miR-34a*	microRNA-34a
*miR-125b*	microRNA-125b
*miR-135a*	microRNA-135a
DNMT3a	DNA methyltransferases
HDAC	Histone deacetylases
Atg9a	Autophagy-related 9A
Snail1	Snail family transcriptional repressor 1
EMT	Epithelial–mesenchymal transition
Bcl-2	B-cell lymphoma 2
PUMA	p53 upregulated modulator of apoptosis
Siah1	Seven in absentia homolog 1
p38 MAPK	P38 mitogen-activated protein kinase
P53	Tumor protein P53
*ACC*	Accuracy
*AUC*	Area under the receiver operating characteristic curve
*PPV*	Positive predictive value
*Recall*	Ratio of correct positive predictions to actual positives
*MCC*	Matthews correlation coefficient
*EstFP*	Estate fingerprint
*MACCS*	MACCS fingerprint
*Pub**c**hemFP*	PubChem fingerprint
*GraphFP*	CDK Graph Only fingerprint
*KRFP*	Klekota–Roth fingerprint
ANN	Multilayer Perceptron -Based Neural Networks
KNN	K-neighbors classifier
GNB	Gaussian naive bayes
RF	Random forest
SVC	Support vector machine
XGB	Extreme gradient boosting decision tree
