# Supplementary material for: Biologically Informed Machine Learning Prioritizes Dietary Supplements That Protect Neural Crest Cells from Ethanol-Induced Epigenetic Dysregulation and Developmental Impairment
Source: Int J Mol Sci. 2025 Dec 27;27(1):295. doi: 10.3390/ijms27010295 (PMC12785273; doi:10.3390/ijms27010295)
Supplement: Supplementary file 1 [file ijms-27-00295-s001.zip › ijms-4012786-supplementary.pdf]

Supplemental information

Biologically informed machine learning prioritizes dietary supplements that protect neural crest cells from ethanol-induced epigenetic dysregulation and developmental impairment

Xiaoqing Wang, Miao Bai, Shuoyang Wang, Hongjia Qian, Jie Liu, Wenke Feng, Huang-ge Zhang, Xiaoyang Wu, Shao-yu Chen

Supplemental table

Table S1: Comparative performance evaluation of the developed machine learning models using MCC, Accuracy (ACC), Area Under the Curve (AUC), F1-score, Positive Predictive Value (PPV), and Recall on both training and external testing sets

MiR-34a															
Training Set								Testing Set							
descriptor	Algorithm	MCC	ACC	AUC	F1	P	Recall	descriptor	Algorithm	MCC	ACC	AUC	F1	P	Recall
Estate	GNB	0.58	0.75	0.75	0.80	0.67	1.00	*Estate*	GNB	-0.58	0.25	0.25	0.40	0.33	0.50
GraghFP	GNB	0.58	0.75	0.75	0.80	0.67	1.00	*ExtFP*	GNB	0.00	0.50	0.50	0.50	0.50	0.50
KRFP	GNB	0.77	0.88	0.88	0.89	0.80	1.00	*KRFP*	GNB	0.00	0.50	0.50	0.00	0.00	0.00
MACCS	GNB	0.67	0.81	0.81	0.84	0.73	1.00	*MACCS*	GNB	-0.58	0.25	0.25	0.00	0.00	0.00
PubchemFP	GNB	0.88	0.94	0.94	0.94	0.89	1.00	*PubchemFP*	GNB	0.00	0.50	0.50	0.50	0.50	0.50
1D & 2D molecular descriptors	GNB	0.48	0.69	0.69	0.55	1.00	0.38	*1D & 2D molecular descriptors*	GNB	0.00	0.50	0.50	0.00	0.00	0.00
Estate	KNN	0.50	0.75	0.75	0.75	0.75	0.75	*Estate*	KNN	0.00	0.50	0.50	0.50	0.50	0.50
GraghFP	KNN	0.50	0.75	0.75	0.75	0.75	0.75	*ExtFP*	KNN	0.00	0.50	0.50	0.00	0.00	0.00
KRFP	KNN	0.26	0.63	0.63	0.67	0.60	0.75	*KRFP*	KNN	0.00	0.50	0.50	0.50	0.50	0.50
MACCS	KNN	0.00	0.50	0.50	0.43	0.50	0.38	*MACCS*	KNN	0.00	0.50	0.50	0.50	0.50	0.50
PubchemFP	KNN	0.13	0.56	0.56	0.63	0.55	0.75	*PubchemFP*	KNN	-1.00	0.00	0.00	0.00	0.00	0.00
1D & 2D molecular descriptors	KNN	0.25	0.63	0.63	0.63	0.63	0.63	*1D & 2D molecular descriptors*	KNN	0.00	0.50	0.50	0.50	0.50	0.50
Estate	ANN	0.88	0.94	0.94	0.94	0.89	1.00	*Estate*	ANN	0.00	0.50	0.50	0.50	0.50	0.50
GraghFP	ANN	0.88	0.94	0.94	0.94	0.89	1.00	*ExtFP*	ANN	0.00	0.50	0.50	0.50	0.50	0.50
KRFP	ANN	0.88	0.94	0.94	0.94	0.89	1.00	*KRFP*	ANN	-0.58	0.25	0.25	0.00	0.00	0.00
MACCS	ANN	0.88	0.94	0.94	0.94	0.89	1.00	*MACCS*	ANN	0.00	0.50	0.50	0.50	0.50	0.50
PubchemFP	ANN	0.88	0.94	0.94	0.93	1.00	0.88	*PubchemFP*	ANN	0.00	0.50	0.50	0.50	0.50	0.50

1D & 2D molecular descriptors	ANN	0.88	0.94	0.94	0.94	0.89	1.00	*1D & 2D molecular descriptors*	ANN	0.58	0.75	0.75	0.67	1.00	0.50
Estate	RF	0.88	0.94	0.94	0.94	0.89	1.00	*Estate*	RF	0.00	0.50	0.50	0.50	0.50	0.50
GraghFP	RF	0.88	0.94	0.94	0.94	0.89	1.00	*ExtFP*	RF	0.00	0.50	0.50	0.50	0.50	0.50
KRFP	RF	0.88	0.94	0.94	0.93	1.00	0.88	*KRFP*	RF	0.00	0.50	0.50	0.50	0.50	0.50
MACCS	RF	0.88	0.94	0.94	0.94	0.89	1.00	*MACCS*	RF	0.00	0.50	0.50	0.50	0.50	0.50
PubchemFP	RF	0.88	0.94	0.94	0.93	1.00	0.88	*PubchemFP*	RF	0.00	0.50	0.50	0.50	0.50	0.50
1D & 2D molecular descriptors	RF	1.00	1.00	1.00	1.00	1.00	1.00	*1D & 2D molecular descriptors*	RF	0.00	0.50	0.50	0.50	0.50	0.50
Estate	SVC	0.88	0.94	0.94	0.94	0.89	1.00	*Estate*	SVC	0.00	0.50	0.50	0.50	0.50	0.50
GraghFP	SVC	0.63	0.81	0.81	0.80	0.86	0.75	*ExtFP*	SVC	-0.58	0.25	0.25	0.00	0.00	0.00
KRFP	SVC	0.77	0.88	0.88	0.89	0.80	1.00	*KRFP*	SVC	0.58	0.75	0.75	0.80	0.67	1.00
MACCS	SVC	0.88	0.94	0.94	0.94	0.89	1.00	*MACCS*	SVC	0.00	0.50	0.50	0.50	0.50	0.50
PubchemFP	SVC	0.77	0.88	0.88	0.89	0.80	1.00	*PubchemFP*	SVC	0.00	0.50	0.50	0.50	0.50	0.50
1D & 2D molecular descriptors	SVC	0.16	0.56	0.56	0.36	0.67	0.25	*1D & 2D molecular descriptors*	SVC	0.00	0.50	0.50	0.00	0.00	0.00
Estate	XGB	0.88	0.94	0.94	0.93	1.00	0.88	*Estate*	XGB	-0.58	0.25	0.25	0.40	0.33	0.50
GraghFP	XGB	0.67	0.81	0.81	0.84	0.73	1.00	*ExtFP*	XGB	0.58	0.75	0.75	0.80	0.67	1.00
KRFP	XGB	0.77	0.88	0.88	0.86	1.00	0.75	*KRFP*	XGB	1.00	1.00	1.00	1.00	1.00	1.00
MACCS	XGB	0.75	0.88	0.88	0.88	0.88	0.88	*MACCS*	XGB	0.58	0.75	0.75	0.67	1.00	0.50
PubchemFP	XGB	0.75	0.88	0.88	0.88	0.88	0.88	*PubchemFP*	XGB	0.58	0.75	0.75	0.67	1.00	0.50
1D & 2D molecular descriptors	XGB	1.00	1.00	1.00	1.00	1.00	1.00	*1D & 2D molecular descriptors*	XGB	0.00	0.50	0.50	0.00	0.00	0.00
DNMT3a															
Training Set								Testing Set							
descriptor	Algorithm	MCC	ACC	AUC	F1	P	Recall	descriptor	Algorithm	MCC	ACC	AUC	F1	P	Recall

Estate	GNB	0.36	0.61	0.62	0.39	1.00	0.24	*Estate*	GNB	0.09	0.57	0.54	0.40	0.50	0.33
GraghFP	GNB	0.66	0.80	0.80	0.84	0.73	1.00	*ExtFP*	GNB	-0.25	0.36	0.40	0.47	0.36	0.67
KRFP	GNB	0.77	0.88	0.87	0.89	0.81	1.00	*KRFP*	GNB	0.10	0.50	0.54	0.59	0.45	0.83
MACCS	GNB	0.51	0.71	0.70	0.78	0.64	1.00	*MACCS*	GNB	-0.25	0.36	0.40	0.47	0.36	0.67
PubchemFP	GNB	0.68	0.82	0.81	0.85	0.74	1.00	*PubchemFP*	GNB	-0.25	0.36	0.40	0.47	0.36	0.67
1D & 2D molecular descriptors	GNB	0.39	0.70	0.69	0.72	0.69	0.76	*1D & 2D molecular descriptors*	GNB	0.24	0.50	0.56	0.63	0.46	1.00
Estate	KNN	0.46	0.73	0.73	0.75	0.72	0.79	*Estate*	KNN	0.17	0.57	0.58	0.57	0.50	0.67
GraghFP	KNN	0.25	0.63	0.62	0.69	0.61	0.79	*ExtFP*	KNN	0.10	0.50	0.54	0.59	0.45	0.83
KRFP	KNN	0.55	0.77	0.76	0.80	0.72	0.90	*KRFP*	KNN	0.04	0.50	0.52	0.53	0.44	0.67
MACCS	KNN	0.21	0.61	0.60	0.66	0.60	0.72	*MACCS*	KNN	-0.41	0.29	0.31	0.37	0.30	0.50
PubchemFP	KNN	0.33	0.66	0.65	0.72	0.63	0.83	*PubchemFP*	KNN	-0.06	0.43	0.48	0.56	0.42	0.83
1D & 2D molecular descriptors	KNN	0.47	0.73	0.73	0.73	0.77	0.69	*1D & 2D molecular descriptors*	KNN	-0.29	0.36	0.35	0.31	0.29	0.33
Estate	ANN	0.86	0.93	0.93	0.93	0.96	0.90	*Estate*	ANN	0.17	0.57	0.58	0.57	0.50	0.67
GraghFP	ANN	0.86	0.93	0.93	0.93	0.90	0.97	*ExtFP*	ANN	0.34	0.64	0.67	0.67	0.56	0.83
KRFP	ANN	0.86	0.93	0.93	0.93	0.93	0.93	*KRFP*	ANN	0.04	0.50	0.52	0.53	0.44	0.67
MACCS	ANN	0.86	0.93	0.93	0.93	0.96	0.90	*MACCS*	ANN	-0.13	0.43	0.44	0.43	0.38	0.50
PubchemFP	ANN	0.86	0.93	0.93	0.93	0.90	0.97	*PubchemFP*	ANN	0.04	0.50	0.52	0.53	0.44	0.67
1D & 2D molecular descriptors	ANN	0.71	0.86	0.86	0.86	0.86	0.86	*1D & 2D molecular descriptors*	ANN	0.10	0.50	0.54	0.59	0.45	0.83
Estate	RF	0.86	0.93	0.93	0.93	0.96	0.90	*Estate*	RF	0.17	0.57	0.58	0.57	0.50	0.67
GraghFP	RF	0.86	0.93	0.93	0.93	0.93	0.93	*ExtFP*	RF	0.17	0.57	0.58	0.57	0.50	0.67
KRFP	RF	0.86	0.93	0.93	0.93	0.90	0.97	*KRFP*	RF	0.10	0.50	0.54	0.59	0.45	0.83
MACCS	RF	0.86	0.93	0.93	0.93	0.93	0.93	*MACCS*	RF	-0.09	0.43	0.46	0.50	0.40	0.67
PubchemFP	RF	0.86	0.93	0.93	0.93	0.96	0.90	*PubchemFP*	RF	0.04	0.50	0.52	0.53	0.44	0.67

1D & 2D molecular descriptors	RF	0.86	0.93	0.93	0.93	0.93	0.93	*1D & 2D molecular descriptors*	RF	0.04	0.50	0.52	0.53	0.44	0.67
Estate	SVC	0.65	0.82	0.82	0.84	0.79	0.90	*Estate*	SVC	0.23	0.57	0.60	0.63	0.50	0.83
GraghFP	SVC	0.52	0.75	0.74	0.79	0.70	0.90	*ExtFP*	SVC	0.10	0.50	0.54	0.59	0.45	0.83
KRFP	SVC	0.72	0.86	0.85	0.87	0.82	0.93	*KRFP*	SVC	-0.25	0.36	0.40	0.47	0.36	0.67
MACCS	SVC	0.70	0.84	0.83	0.86	0.78	0.97	*MACCS*	SVC	-0.41	0.29	0.31	0.37	0.30	0.50
PubchemFP	SVC	0.52	0.73	0.72	0.79	0.67	0.97	*PubchemFP*	SVC	0.10	0.50	0.54	0.59	0.45	0.83
1D & 2D molecular descriptors	SVC	0.23	0.61	0.60	0.69	0.58	0.86	*1D & 2D molecular descriptors*	SVC	0.00	0.43	0.50	0.60	0.43	1.00
Estate	XGB	0.79	0.89	0.89	0.90	0.90	0.90	*Estate*	XGB	0.26	0.64	0.63	0.55	0.60	0.50
GraghFP	XGB	0.79	0.89	0.89	0.90	0.87	0.93	*ExtFP*	XGB	0.29	0.64	0.65	0.62	0.57	0.67
KRFP	XGB	0.79	0.89	0.89	0.90	0.90	0.90	*KRFP*	XGB	0.41	0.71	0.69	0.60	0.75	0.50
MACCS	XGB	0.86	0.93	0.93	0.93	0.93	0.93	*MACCS*	XGB	0.04	0.50	0.52	0.53	0.44	0.67
PubchemFP	XGB	0.82	0.91	0.91	0.91	0.93	0.90	*PubchemFP*	XGB	0.17	0.57	0.58	0.57	0.50	0.67
1D & 2D molecular descriptors	XGB	0.87	0.93	0.93	0.93	1.00	0.86	*1D & 2D molecular descriptors*		0.17	0.57	0.58	0.57	0.50	0.67
HDAC													
Training Set								Testing Set					
descriptor	Algorithm	MCC	ACC	AUC	F1	P	Recall	descriptor	Algorithm	MCC	ACC	AUC	F1	P	Recall
Estate	GNB	0.76	0.87	0.87	0.85	1.00	0.73	*Estate*	GNB	0.77	0.88	0.88	0.86	1.00	0.75
GraghFP	GNB	0.94	0.97	0.97	0.97	1.00	0.93	*ExtFP*	GNB	0.26	0.63	0.63	0.67	0.60	0.75
KRFP	GNB	1.00	1.00	1.00	1.00	1.00	1.00	*KRFP*	GNB	0.58	0.75	0.75	0.80	0.67	1.00
MACCS	GNB	0.60	0.80	0.80	0.80	0.80	0.80	*MACCS*	GNB	0.77	0.88	0.88	0.86	1.00	0.75
PubchemFP	GNB	1.00	1.00	1.00	1.00	1.00	1.00	*PubchemFP*	GNB	1.00	1.00	1.00	1.00	1.00	1.00
1D & 2D molecular descriptors	GNB	0.53	0.77	0.77	0.77	0.75	0.80	*1D & 2D molecular descriptors*	GNB	0.58	0.75	0.75	0.80	0.67	1.00

Estate	KNN	0.36	0.67	0.67	0.58	0.78	0.47	*Estate*	KNN	0.77	0.88	0.88	0.86	1.00	0.75
GraghFP	KNN	0.61	0.80	0.80	0.79	0.85	0.73	*ExtFP*	KNN	0.77	0.88	0.88	0.86	1.00	0.75
KRFP	KNN	0.51	0.73	0.73	0.78	0.67	0.93	*KRFP*	KNN	0.00	0.50	0.50	0.67	0.50	1.00
MACCS	KNN	0.53	0.77	0.77	0.77	0.75	0.80	*MACCS*	KNN	0.58	0.75	0.75	0.67	1.00	0.50
PubchemFP	KNN	0.94	0.97	0.97	0.97	1.00	0.93	*PubchemFP*	KNN	1.00	1.00	1.00	1.00	1.00	1.00
1D & 2D molecular descriptors	KNN	0.40	0.70	0.70	0.69	0.71	0.67	*1D & 2D molecular descriptors*	KNN	0.26	0.63	0.63	0.67	0.60	0.75
Estate	ANN	1.00	1.00	1.00	1.00	1.00	1.00	*Estate*	ANN	0.58	0.75	0.75	0.80	0.67	1.00
GraghFP	ANN	1.00	1.00	1.00	1.00	1.00	1.00	*ExtFP*	ANN	1.00	1.00	1.00	1.00	1.00	1.00
KRFP	ANN	1.00	1.00	1.00	1.00	1.00	1.00	*KRFP*	ANN	0.58	0.75	0.75	0.80	0.67	1.00
MACCS	ANN	1.00	1.00	1.00	1.00	1.00	1.00	*MACCS*	ANN	0.26	0.63	0.63	0.67	0.60	0.75
PubchemFP	ANN	1.00	1.00	1.00	1.00	1.00	1.00	*PubchemFP*	ANN	1.00	1.00	1.00	1.00	1.00	1.00
1D & 2D molecular descriptors	ANN	0.80	0.90	0.90	0.90	0.93	0.87	*1D & 2D molecular descriptors*	ANN	0.00	0.50	0.50	0.50	0.50	0.50
Estate	RF	1.00	1.00	1.00	1.00	1.00	1.00	*Estate*	RF	1.00	1.00	1.00	1.00	1.00	1.00
GraghFP	RF	1.00	1.00	1.00	1.00	1.00	1.00	*ExtFP*	RF	1.00	1.00	1.00	1.00	1.00	1.00
KRFP	RF	1.00	1.00	1.00	1.00	1.00	1.00	*KRFP*	RF	0.58	0.75	0.75	0.80	0.67	1.00
MACCS	RF	1.00	1.00	1.00	1.00	1.00	1.00	*MACCS*	RF	0.77	0.88	0.88	0.86	1.00	0.75
PubchemFP	RF	1.00	1.00	1.00	1.00	1.00	1.00	*PubchemFP*	RF	1.00	1.00	1.00	1.00	1.00	1.00
1D & 2D molecular descriptors	RF	1.00	1.00	1.00	1.00	1.00	1.00	*1D & 2D molecular descriptors*	RF	1.00	1.00	1.00	1.00	1.00	1.00
Estate	SVC	0.71	0.83	0.83	0.80	1.00	0.67	*Estate*	SVC	0.77	0.88	0.88	0.86	1.00	0.75
GraghFP	SVC	0.67	0.83	0.83	0.84	0.81	0.87	*ExtFP*	SVC	1.00	1.00	1.00	1.00	1.00	1.00
KRFP	SVC	1.00	1.00	1.00	1.00	1.00	1.00	*KRFP*	SVC	0.26	0.63	0.63	0.67	0.60	0.75
MACCS	SVC	0.94	0.97	0.97	0.97	1.00	0.93	*MACCS*	SVC	0.58	0.75	0.75	0.67	1.00	0.50
PubchemFP	SVC	0.94	0.97	0.97	0.97	1.00	0.93	*PubchemFP*	SVC	1.00	1.00	1.00	1.00	1.00	1.00

1D & 2D molecular descriptors	SVC	0.27	0.63	0.63	0.62	0.64	0.60	*1D & 2D molecular descriptors*	SVC	-0.38	0.38	0.38	0.55	0.43	0.75
Estate	XGB	0.94	0.97	0.97	0.97	1.00	0.93	*Estate*	XGB	1.00	1.00	1.00	1.00	1.00	1.00
GraghFP	XGB	0.87	0.93	0.93	0.93	0.93	0.93	*ExtFP*	XGB	0.77	0.88	0.88	0.86	1.00	0.75
KRFP	XGB	1.00	1.00	1.00	1.00	1.00	1.00	*KRFP*	XGB	0.58	0.75	0.75	0.80	0.67	1.00
MACCS	XGB	1.00	1.00	1.00	1.00	1.00	1.00	*MACCS*	XGB	0.58	0.75	0.75	0.80	0.67	1.00
PubchemFP	XGB	1.00	1.00	1.00	1.00	1.00	1.00	*PubchemFP*	XGB	1.00	1.00	1.00	1.00	1.00	1.00
1D & 2D molecular descriptors	XGB	1.00	1.00	1.00	1.00	1.00	1.00	*1D & 2D molecular descriptors*		0.77	0.88	0.88	0.86	1.00	0.75
MiR-125b															
Training Set								Testing Set							
descriptor	Algorithm	MCC	ACC	AUC	F1	P	Recall	descriptor	Algorithm	MCC	ACC	AUC	F1	P	Recall
Estate	GNB	0.49	0.67	0.72	0.62	1.00	0.44	*Estate*	GNB	0.00	0.75	0.50	0.00	0.00	0.00
GraghFP	GNB	0.76	0.87	0.89	0.88	1.00	0.78	*ExtFP*	GNB	0.58	0.75	0.83	0.67	0.50	1.00
KRFP	GNB	0.87	0.93	0.92	0.95	0.90	1.00	*KRFP*	GNB	-1.00	0.00	0.00	0.00	0.00	0.00
MACCS	GNB	0.76	0.87	0.89	0.88	1.00	0.78	*MACCS*	GNB	-0.33	0.50	0.33	0.00	0.00	0.00
PubchemFP	GNB	0.76	0.87	0.89	0.88	1.00	0.78	*PubchemFP*	GNB	-0.33	0.50	0.33	0.00	0.00	0.00
1D & 2D molecular descriptors	GNB	0.61	0.80	0.75	0.86	0.75	1.00	*1D & 2D molecular descriptors*	GNB	0.00	0.25	0.50	0.40	0.25	1.00
Estate	KNN	0.27	0.67	0.61	0.76	0.67	0.89	*Estate*	KNN	-0.33	0.50	0.33	0.00	0.00	0.00
GraghFP	KNN	0.43	0.73	0.69	0.80	0.73	0.89	*ExtFP*	KNN	0.33	0.50	0.67	0.50	0.33	1.00
KRFP	KNN	0.48	0.73	0.67	0.82	0.69	1.00	*KRFP*	KNN	0.00	0.25	0.50	0.40	0.25	1.00
MACCS	KNN	0.08	0.60	0.53	0.73	0.62	0.89	*MACCS*	KNN	0.00	0.25	0.50	0.40	0.25	1.00
PubchemFP	KNN	0.48	0.73	0.67	0.82	0.69	1.00	*PubchemFP*	KNN	0.00	0.25	0.50	0.40	0.25	1.00
1D & 2D molecular descriptors	KNN	0.33	0.67	0.58	0.78	0.64	1.00	*1D & 2D molecular descriptors*	KNN	0.00	0.25	0.50	0.40	0.25	1.00

Estate	ANN	1.00	1.00	1.00	1.00	1.00	1.00	*Estate*	ANN	0.33	0.50	0.67	0.50	0.33	1.00
GraghFP	ANN	1.00	1.00	1.00	1.00	1.00	1.00	*ExtFP*	ANN	0.58	0.75	0.83	0.67	0.50	1.00
KRFP	ANN	1.00	1.00	1.00	1.00	1.00	1.00	*KRFP*	ANN	-0.33	0.50	0.33	0.00	0.00	0.00
MACCS	ANN	1.00	1.00	1.00	1.00	1.00	1.00	*MACCS*	ANN	0.33	0.50	0.67	0.50	0.33	1.00
PubchemFP	ANN	1.00	1.00	1.00	1.00	1.00	1.00	*PubchemFP*	ANN	-0.33	0.50	0.33	0.00	0.00	0.00
1D & 2D molecular descriptors	ANN	0.32	0.53	0.61	0.36	1.00	0.22	*1D & 2D molecular descriptors*	ANN	-0.58	0.25	0.17	0.00	0.00	0.00
Estate	RF	1.00	1.00	1.00	1.00	1.00	1.00	*Estate*	RF	0.33	0.50	0.67	0.50	0.33	1.00
GraghFP	RF	1.00	1.00	1.00	1.00	1.00	1.00	*ExtFP*	RF	0.58	0.75	0.83	0.67	0.50	1.00
KRFP	RF	1.00	1.00	1.00	1.00	1.00	1.00	*KRFP*	RF	0.58	0.75	0.83	0.67	0.50	1.00
MACCS	RF	1.00	1.00	1.00	1.00	1.00	1.00	*MACCS*	RF	0.33	0.50	0.67	0.50	0.33	1.00
PubchemFP	RF	1.00	1.00	1.00	1.00	1.00	1.00	*PubchemFP*	RF	0.33	0.50	0.67	0.50	0.33	1.00
1D & 2D molecular descriptors	RF	1.00	1.00	1.00	1.00	1.00	1.00	*1D & 2D molecular descriptors*	RF	0.00	0.25	0.50	0.40	0.25	1.00
Estate	SVC	0.74	0.87	0.83	0.90	0.82	1.00	*Estate*	SVC	0.00	0.25	0.50	0.40	0.25	1.00
GraghFP	SVC	0.61	0.80	0.75	0.86	0.75	1.00	*ExtFP*	SVC	0.00	0.25	0.50	0.40	0.25	1.00
KRFP	SVC	0.74	0.87	0.83	0.90	0.82	1.00	*KRFP*	SVC	0.00	0.25	0.50	0.40	0.25	1.00
MACCS	SVC	0.87	0.93	0.92	0.95	0.90	1.00	*MACCS*	SVC	0.00	0.25	0.50	0.40	0.25	1.00
PubchemFP	SVC	0.74	0.87	0.83	0.90	0.82	1.00	*PubchemFP*	SVC	0.00	0.25	0.50	0.40	0.25	1.00
1D & 2D molecular descriptors	SVC	0.48	0.73	0.67	0.82	0.69	1.00	*1D & 2D molecular descriptors*	SVC	0.00	0.25	0.50	0.40	0.25	1.00
Estate	XGB	1.00	1.00	1.00	1.00	1.00	1.00	*Estate*	XGB	0.58	0.75	0.83	0.67	0.50	1.00
GraghFP	XGB	0.44	0.73	0.72	0.78	0.78	0.78	*ExtFP*	XGB	0.33	0.50	0.67	0.50	0.33	1.00
KRFP	XGB	0.74	0.87	0.83	0.90	0.82	1.00	*KRFP*	XGB	-0.58	0.25	0.17	0.00	0.00	0.00
MACCS	XGB	0.87	0.93	0.92	0.95	0.90	1.00	*MACCS*	XGB	0.33	0.50	0.67	0.50	0.33	1.00
PubchemFP	XGB	1.00	1.00	1.00	1.00	1.00	1.00	*PubchemFP*	XGB	0.58	0.75	0.83	0.67	0.50	1.00

1D & 2D molecular descriptors	XGB	1.00	1.00	1.00	1.00	1.00	1.00	*1D & 2D molecular descriptors*	XGB	0.00	0.25	0.50	0.40	0.25	1.00
MiR-135a															
Training Set								Testing Set							
descriptor	Algorithm	MCC	ACC	AUC	F1	P	Recall	descriptor	Algorithm	MCC	ACC	AUC	F1	P	Recall
Estate	GNB	1.00	1.00	1.00	1.00	1.00	1.00	*Estate*	GNB	0.00	0.50	0.50	0.00	0.00	0.00
GraghFP	GNB	1.00	1.00	1.00	1.00	1.00	1.00	*ExtFP*	GNB	1.00	1.00	1.00	1.00	1.00	1.00
KRFP	GNB	1.00	1.00	1.00	1.00	1.00	1.00	*KRFP*	GNB	0.00	0.50	0.50	0.00	0.00	0.00
MACCS	GNB	1.00	1.00	1.00	1.00	1.00	1.00	*MACCS*	GNB	0.00	0.50	0.50	0.00	0.00	0.00
PubchemFP	GNB	1.00	1.00	1.00	1.00	1.00	1.00	*PubchemFP*	GNB	0.00	0.50	0.50	0.00	0.00	0.00
1D & 2D molecular descriptors	GNB	1.00	1.00	1.00	1.00	1.00	1.00	*1D & 2D molecular descriptors*	GNB	0.00	0.50	0.50	0.00	0.00	0.00
Estate	KNN	###	0.43	0.38	0.00	0.00	0.00	*Estate*	KNN	0.00	0.50	0.50	0.67	0.50	1.00
GraghFP	KNN	0.55	0.71	0.75	0.75	0.60	1.00	*ExtFP*	KNN	0.00	0.50	0.50	0.67	0.50	1.00
KRFP	KNN	0.42	0.71	0.71	0.67	0.67	0.67	*KRFP*	KNN	-1.00	0.00	0.00	0.00	0.00	0.00
MACCS	KNN	###	0.43	0.38	0.00	0.00	0.00	*MACCS*	KNN	0.00	0.50	0.50	0.00	0.00	0.00
PubchemFP	KNN	0.09	0.57	0.54	0.40	0.50	0.33	*PubchemFP*	KNN	0.00	0.50	0.50	0.67	0.50	1.00
1D & 2D molecular descriptors	KNN	0.00	0.57	0.50	0.00	0.00	0.00	*1D & 2D molecular descriptors*	KNN	0.00	0.50	0.50	0.00	0.00	0.00
Estate	ANN	1.00	1.00	1.00	1.00	1.00	1.00	*Estate*	ANN	0.00	0.50	0.50	0.00	0.00	0.00
GraghFP	ANN	1.00	1.00	1.00	1.00	1.00	1.00	*ExtFP*	ANN	1.00	1.00	1.00	1.00	1.00	1.00
KRFP	ANN	1.00	1.00	1.00	1.00	1.00	1.00	*KRFP*	ANN	-1.00	0.00	0.00	0.00	0.00	0.00
MACCS	ANN	1.00	1.00	1.00	1.00	1.00	1.00	*MACCS*	ANN	0.00	0.50	0.50	0.00	0.00	0.00
PubchemFP	ANN	1.00	1.00	1.00	1.00	1.00	1.00	*PubchemFP*	ANN	0.00	0.50	0.50	0.00	0.00	0.00
1D & 2D molecular descriptors	ANN	1.00	1.00	1.00	1.00	1.00	1.00	*1D & 2D molecular descriptors*	ANN	-1.00	0.00	0.00	0.00	0.00	0.00

Estate	RF	1.00	1.00	1.00	1.00	1.00	1.00	*Estate*	RF	0.00	0.50	0.50	0.00	0.00	0.00
GraghFP	RF	1.00	1.00	1.00	1.00	1.00	1.00	*ExtFP*	RF	0.00	0.50	0.50	0.00	0.00	0.00
KRFP	RF	1.00	1.00	1.00	1.00	1.00	1.00	*KRFP*	RF	0.00	0.50	0.50	0.00	0.00	0.00
MACCS	RF	1.00	1.00	1.00	1.00	1.00	1.00	*MACCS*	RF	0.00	0.50	0.50	0.00	0.00	0.00
PubchemFP	RF	1.00	1.00	1.00	1.00	1.00	1.00	*PubchemFP*	RF	0.00	0.50	0.50	0.67	0.50	1.00
1D & 2D molecular descriptors	RF	1.00	1.00	1.00	1.00	1.00	1.00	*1D & 2D molecular descriptors*	RF	-1.00	0.00	0.00	0.00	0.00	0.00
Estate	SVC	1.00	1.00	1.00	1.00	1.00	1.00	*Estate*	SVC	0.00	0.50	0.50	0.00	0.00	0.00
GraghFP	SVC	1.00	1.00	1.00	1.00	1.00	1.00	*ExtFP*	SVC	0.00	0.50	0.50	0.00	0.00	0.00
KRFP	SVC	0.73	0.86	0.83	0.80	1.00	0.67	*KRFP*	SVC	0.00	0.50	0.50	0.00	0.00	0.00
MACCS	SVC	1.00	1.00	1.00	1.00	1.00	1.00	*MACCS*	SVC	0.00	0.50	0.50	0.00	0.00	0.00
PubchemFP	SVC	1.00	1.00	1.00	1.00	1.00	1.00	*PubchemFP*	SVC	0.00	0.50	0.50	0.00	0.00	0.00
1D & 2D molecular descriptors	SVC	0.73	0.86	0.83	0.80	1.00	0.67	*1D & 2D molecular descriptors*	SVC	-1.00	0.00	0.00	0.00	0.00	0.00
Estate	XGB	0.00	0.57	0.50	0.00	0.00	0.00	*Estate*	XGB	0.00	0.50	0.50	0.00	0.00	0.00
GraghFP	XGB	0.00	0.57	0.50	0.00	0.00	0.00	*ExtFP*	XGB	0.00	0.50	0.50	0.00	0.00	0.00
KRFP	XGB	0.00	0.57	0.50	0.00	0.00	0.00	*KRFP*	XGB	0.00	0.50	0.50	0.00	0.00	0.00
MACCS	XGB	0.00	0.57	0.50	0.00	0.00	0.00	*MACCS*	XGB	0.00	0.50	0.50	0.00	0.00	0.00
PubchemFP	XGB	0.00	0.57	0.50	0.00	0.00	0.00	*PubchemFP*	XGB	0.00	0.50	0.50	0.00	0.00	0.00
1D & 2D molecular descriptors	XGB	0.00	0.57	0.50	0.00	0.00	0.00	*1D & 2D molecular descriptors*	XGB	0.00	0.50	0.50	0.00	0.00	0.00

Notes: ACC: Accuracy; AUC: Area under the receiver operating characteristic curve; PPV: Positive predictive value; Recall: Ratio of correct positive predictions to actual positives; F1: F1 Score; MCC: Matthews correlation coefficient; ANN-MLP: neural networks - multilayer perceptron; KNN: K-neighbors classifier; GNB: Gaussian naive bayes; RF: Random forest; SVC: Support vector machine, XGB: Extreme gradient boosting decision tree; PubchemFP: Publicly available fingerprint descriptors capturing presence of substructures; MACCS: 166-bit structural key fingerprints, commonly used in cheminformatics; KRFP: Klekota-Roth fingerprint, substructure-based fingerprint

encoding the presence of predefined chemical fragments; GraphFP: Graph-based fingerprints capturing topological features; EstFP: Electrotopological state indices reflecting electronic and topological properties.
